# Quercetin and *Camellia sinensis* leaf extract ameliorates liver steatosis in high-fat diet fed mice via suppression of oxidative stress and inflammation

**DOI:** 10.22038/ajp.2025.26208

**Published:** 2025

**Authors:** Mahdieh Sadat Badiee, Ali Vadizadeh, Maryam Salehcheh, Mehrnoosh Moosavi, Fereshtesadat Fakhredini, Hadi Kalantar, Sirous Rafiei Asl, Mohammad Javad Khodayar

**Affiliations:** 1 *Student Research Committee, Ahvaz Jundishapur University of Medical Sciences, Ahvaz, Iran*; 2 *Department of Toxicology, Faculty of Pharmacy, Ahvaz Jundishapur University of Medical Sciences, Ahvaz, Iran.*; 3 *Toxicology Research Center, Medical Basic Sciences Research Institute, Ahvaz Jundishapur University of Medical Sciences, Ahvaz, Iran *; 4 *Cellular and Molecular Research Center, Medical Basic Sciences Research Institute, Ahvaz Jundishapur University of Medical Sciences, Ahvaz, Iran*; 5 *Cancer, Environmental and Petroleum Pollutants Research Center, Ahvaz Jundishapur University of Medical Sciences, Ahvaz, Iran*

**Keywords:** Quercetin, Camellia sinensis leaf extract High-fat diet, Liver steatosis, Mice

## Abstract

**Objective:**

Fatty liver disease is characterized by excessive fat accumulation in liver tissue, which can lead to liver failure and cirrhosis. Quercetin (QCT) is a flavonoid known for its antioxidant properties. The therapeutic benefits of *Camellia sinensis* leaf extract (CSLE) have been demonstrated in prevention and treatment of various diseases. This research sought to assess the synergistic impact of QCT and CSLE on reduction of liver steatosis in high-fat diet (HFD)-fed mice.

**Materials and Methods:**

Thirty mice were randomized in five groups (n=6), including: control, HFD (10 ml/kg), HFD and QCT (50 mg/kg), HFD and CSLE 2% (200 mg/kg), and HFD and QCT (50 mg/kg) in combination with CSLE 2% (200 mg/kg) in the last week. Mice underwent anesthesia on day 43 after a night of fasting, and the levels of hepatic enzymes and biomarkers, antioxidants, and pro-inflammatory factors were measured.

**Results:**

Treatment with QCT and CSLE reduced serum levels of alkaline phosphatase, alanine aminotransferase, aspartate aminotransferase, cholesterol, triglyceride, low-density lipoprotein cholesterol, very-low-density lipoprotein, total protein, and total bilirubin, and hepatic levels of thiobarbituric acid-reactive substances, while increasing serum high-density lipoprotein cholesterol and the levels of hepatic catalase, superoxide dismutase, and glutathione peroxidase.

**Conclusion:**

Treatment with QCT and CSLE may effectively reduce liver steatosis in HFD-fed mice by improving lipid profiles and antioxidant status.

## Introduction

Fatty liver disease (FLD) is characterized by excessive accumulation of fat within liver cells, which can disrupt normal liver function and potentially result in serious health issues, including liver failure or cirrhosis. Fat accumulation in the liver can occur due to increased fat synthesis or decreased fat excretion and oxidation (Paschos and Paletas 2009). FLD is primarily classified into two categories: alcoholic fatty liver disease and non-alcoholic fatty liver disease (NAFLD) (Williams, et al. 2011). The cause of alcoholic fatty liver is chronic alcohol consumption. Non-alcoholic fatty liver disease is a metabolic disorder that is common in middle-aged individuals with obesity, hyperlipidemia, and type 2 diabetes. According to previous studies, a high-fat diet (HFD) ultimately leads to liver steatosis (Assy, et al. 2000). Excessive intake of biological lipids such as triglyceride and cholesterol leads to hypertriglyceridemia and hypercholesterolemia (Hokanson 2002; Kametani, et al. 2002). Accumulation of triglyceride in the liver cells occurs due to the esterification of free fatty acids and glycerol. Three sources of fat lipolysis, HFD, and re-lipogenesis lead to increased liver free fatty acids (Postic and Girard 2008). Donnelly et al. showed that 60% of triglyceride content originates from adipose tissue lipolysis, 15% from diet, and 26% from re-lipogenesis (Donnelly, et al. 2005). NAFLD is associated with a spectrum of histopathological changes ranging from steatosis to cirrhosis (Angulo 2002; Farrell 2003). Steatosis was previously considered a benign disease. Today, it is known that oxidative stress can induce steatohepatitis and manifest as necrosis, fibrosis, and cirrhosis (Orrenius, et al. 2007). Pharmacological interventions for steatosis include the use of antioxidants, insulin sensitizers, hepatoprotective agents, and lipid-lowering drugs (Comar and Sterling 2006). These methods only treat or control the underlying risk factors. 

Green tea (*Camellia sinensis*) is one of the most popular drinks in the world and is considered a beneficial source for human health today. The therapeutic properties of *C. sinensis* leaf extract (CSLE) and its polyphenols, have been proven in prevention and treatment of many diseases (Mandel, et al. 2006; Ostrowska and Skrzydlewska 2006). Polyphenols, particularly catechins, constitute the principal constituents of green tea. The most important catechins found in green tea include epigallocatechin, epicatechin, and epicatechin gallate. Research by Sano et al. indicated that green tea catechins inhibit lipid peroxidation in liver and kidney tissues (Sano, et al. 1995). Green tea catechins are highly effective in neutralizing superoxide, hydrogen peroxide, hydroxyl radicals, and nitric oxide (NO). Their cathodic structure allows catechins to bind with metals, thereby inhibiting the generation of free radicals (Rice-Evans et al., 1997) (Rice-Evans and Miller 1997). Furthermore, these catechins exhibit antioxidant capabilities that safeguard cellular integrity (Pietta et al., 1998) (Pietta, et al. 1998). Among the teas consumed, green tea has the highest amount of catechin. Catechin can also be found in various foods, including apples, berries, and cocoa in lower concentrations compared to green tea. Given this information, it is plausible that this medicinal plant possesses the capability to safeguard the liver from steatosis and damage caused by oxidative stress. 

Quercetin (QCT) is recognized as the most essential compound within the flavonoid family (Vollmannová, et al. 2024), noted for its remarkable antioxidant properties. This compound is present in vegetables, fruits, onion, apple, red grape, broccoli, tomato, and dark chocolate (Chen 2010; Vieira, et al. 2011). Research has shown that QCT offers protective effects to the liver, heart, kidney, and neurons against oxidant agents (Abarikwu 2014; Abo-Salem, et al. 2011; Zhang, et al. 2011). QCT has anti-inflammatory, antioxidant, anticancer, antiviral, antibacterial, antiallergic, and antihypertensive properties (Cuevas, et al. 2011). It enhances the level of glutathione while simultaneously lowering malondialdehyde level, nitric oxide metabolism, and superoxide production, which collectively contribute to a reduction in oxidative mediators and inflammation (Del Prete, et al. 2012). Furthermore, this compound mitigates the elevation of liver enzymes by alleviating oxidative damage within the liver (Raygude et al., 2012; Coballase-Urrutia et al., 2013) (Coballase-Urrutia, et al. 2013; Raygude, et al. 2012).

The antioxidant properties of QCT and CSLE, as indicated by numerous studies, suggest that it may significantly mitigate oxidative stress and steatosis of the liver. This research aimed to explore the synergistic effects of QCT and CSLE against liver steatosis in HFD through suppression of oxidative stress and inflammation in mice.

## Materials and Methods

### Chemicals

Quercetin (CAS: 117-39-5) and DTNB (CAS: 69-78-3) were acquired from Sigma Aldrich (USA). The kits for analyzing cholesterol (Cho), triglyceride (TG), low-density lipoprotein cholesterol (LDL-C), high-density lipoprotein cholesterol (HDL-C), alkaline phosphatase (ALP), alanine aminotransferase (ALT), aspartate aminotransferase (AST), total bilirubin (T.Bil), and total protein (T.P) were sourced from Pars Azmoon Company (Iran). Superoxide dismutase (SOD) and glutathione peroxidase (GPx) assay kits were acquired from ZellBio (Germany). The tumor necrosis factor alpha (TNF-α) kit was obtained from Bioassay Technology Laboratory (China).

### Preparation of HFD emulsion and C. sinensis leaf extract

The HFD contained ten components emulsified in distilled water. Following its preparation, the mixture was stored at 4 °C, subsequently heated in a water bath, and thoroughly mixed prior to administration. The ingredients utilized in this emulsion are detailed in Table 1 (Zou, et al. 2006). The hydroalcoholic extract of *C. sinensis* leaf (Source of National Genetic Resources Plant Museum, ROC, Collection Date 2023/04/23, Certificate Number 000834074) was prepared using the soaking method. In this process, 300 g of dried plant powder was placed in an Erlenmeyer flask, and 70% ethanol was added until it reached a level 2 cm above the plant material. The mixture was allowed to sit for 72 hr, with stirring conducted three times daily. The extract underwent an initial filtration process utilizing three layers of sterile gauze, followed by filtration through Whatman N. 0.1 paper. Subsequently, a rotary device was employed at 30 °C to concentrate the extract. After concentration, the extract was subjected to freeze drying for a duration of 48 hr to remove moisture. The resulting dry extract was then weighed and appropriately stored in a suitable container.

### Animals

This research involved 30 male NMRI mice, aged 7–8 weeks, with an average weight of 25±2 g. All animals were maintained under identical care conditions. They were exposed to a light/dark cycle of 12 hr each, with a temperature maintained at 28±3 °C, and appropriate relative humidity. The study adhered to all ethical guidelines for the use and care of animals as established by the Institutional Animal Care and Use Committee of Ahvaz Jundishapur University of Medical Sciences (ethics number: IR. AJUMS. ABHC. REC. 1401. 0 53).

### Experimental design

This research was carried out over a period of six weeks. The mice were categorized into five groups (n=6), which included: control (standard diet), HFD (HFD 10 ml/kg, gavage), HFD and QCT (50 mg/kg, gavage), HFD and CSLE 2% (200 mg/kg, gavage), HFD and combination of QCT (50 mg/kg) and CSLE 2% (200 mg/kg) in the last week. The dosages of QCT (Rubio-Ruiz et al., 2019) and CSLE (Karolczak et al., 2019) were determined based on prior studies. Each experimental group was subjected to their respective diets for a six-week duration. Fatty liver induction was performed with high-fat emulsion, the composition of which is detailed in Table 1 (Zou et al., 2006). It was given via gavage every day at 8 am. The animals' weights were meticulously recorded on a weekly basis. At the conclusion of the experiment, the animals were anesthetized using a combination of ketamine (90 mg/kg) and xylazine (10 mg/kg). Blood samples were obtained directly from the heart and kept. The sera were subsequently separated and preserved at –20 °C. Liver tissue was extracted. One portion of the liver was fixed in formalin for histopathological analysis, while another portion was stored at –70 °C for the assessment of oxidative and inflammatory markers.

**Table 1:** *The composition of the high-fat emulsion diet* *(Zou, et al. 2006)* *.*

content	Component
**400**	Corn oil (g)
**150**	Saccharose (g)
**80**	Total milk powder
**100**	Cholesterol (g)
**10**	Sodium deoxycholate (g)
**36.4**	Tween 80 (g)
**31.1**	Propylene glycol (g)
**2.5**	Vitamin mixture (g)
**10**	Cooking salt (g)
**1.5**	Mineral mixture (g)
**300**	Distilled water (ml)

### Body weight and liver weight changes

Mice were weighed weekly, with liver weight assessed after a six-week period. The measurements of liver weight (g) and body weight (g) were documented.

### Biochemical examination

The serum levels of Cho, TG, LDL-C, HDL-C, ALT, AST, ALP, T.Bil, and T.P were assessed utilizing Pars Azmoon assay kits in conjunction with the Hitachi 912 auto-analyzer (Japan). The level of very low-density lipoprotein (VLDL) was determined using the Norbert formula (VLDL-Cho = TG/5) (Tietz et al., 1995).

### Preparation of liver tissue homogenate and determining protein content

A liver tissue sample was homogenized in phosphate- buffered saline (PBS) and centrifuged. The resulting supernatant was collected and preserved at –70 °C for the analysis of tissue factors, including thiobarbituric acid reactive substances (TBARS), SOD, catalase (CAT), GPx, and TNF-α. The protein concentration in the supernatants was assessed using the Bradford protein assay method (He et al., 2011) (He 2011), with absorbance measured at 595 nm.

### Determination of total thiol (TT)

The total thiol level was assessed with Ellman's reagent (DTNB), resulting in the production of yellow TNB (Jafarian, et al. 2013). The absorbance was read at 412 nm and expressed as nmol/mg protein.

### Determination of thiobarbituric acid reactive substances (TBARS)

The level of TBARS was determined utilizing the Satoh et al. method (Satoh 1978) . The absorption of the resulting dye was evaluated at 532 nm and reported as nmol/mg protein.

### Determination of catalase activity

The catalase (CAT) activity was assessed using the Shangari method (Shangari et al., 2006) (Shangari and O'Brien 2006). The absorbance was evaluated at 410 nm, and reported as U/mg protein. 

### Determination of tumor necrosis factor-alpha (TNF-α)

The level of TNF-α was assessed with Bioassay Technology Laboratory kit. The absorbance was read at 450 nm, and reported as pg/mg protein.

### Determination of antioxidant enzymes activity

The activities of the SOD and GPx antioxidant enzymes were assessed calorimetrically utilizing a ZellBio kit, with results expressed as U/mg protein.

### Histopathological examination

A portion of the liver tissue was preserved in 10% formalin. Following preparation of the blocks, thin sections measuring 5 microns in thickness were produced and subsequently stained with the hematoxylin-eosin (H&E) method (Luna and Pathology 1968) . For each specimen, six microscopic slides were examined. Hepatic histological characteristics were classified into four categories: normal (0), mild (1), moderate (2), and severe (3) to assess fatty change in the liver. Grade 0 indicated tissue sections exhibiting a normal structure. Grade 1 reflected a slight level of fatty change. Grade 2 represented a moderate level of fatty change within the liver parenchyma. Grade 3 indicated a significantly high level of fatty change.

### Statistical analysis

Statistical analysis was conducted utilizing Prism software (version 8.0, GraphPad Software, Inc., La Jolla, CA, USA). The normality of the data was verified through the Kolmogorov-Smirnov test. The mean ± SEM was computed, and the results were subsequently evaluated with one-way ANOVA and Tukey's post hoc test for multiple comparisons. A P<0.05 was considered significant.

## Results

### The effect of QCT and CSLE on liver weight and body weight

**Figure 1:**
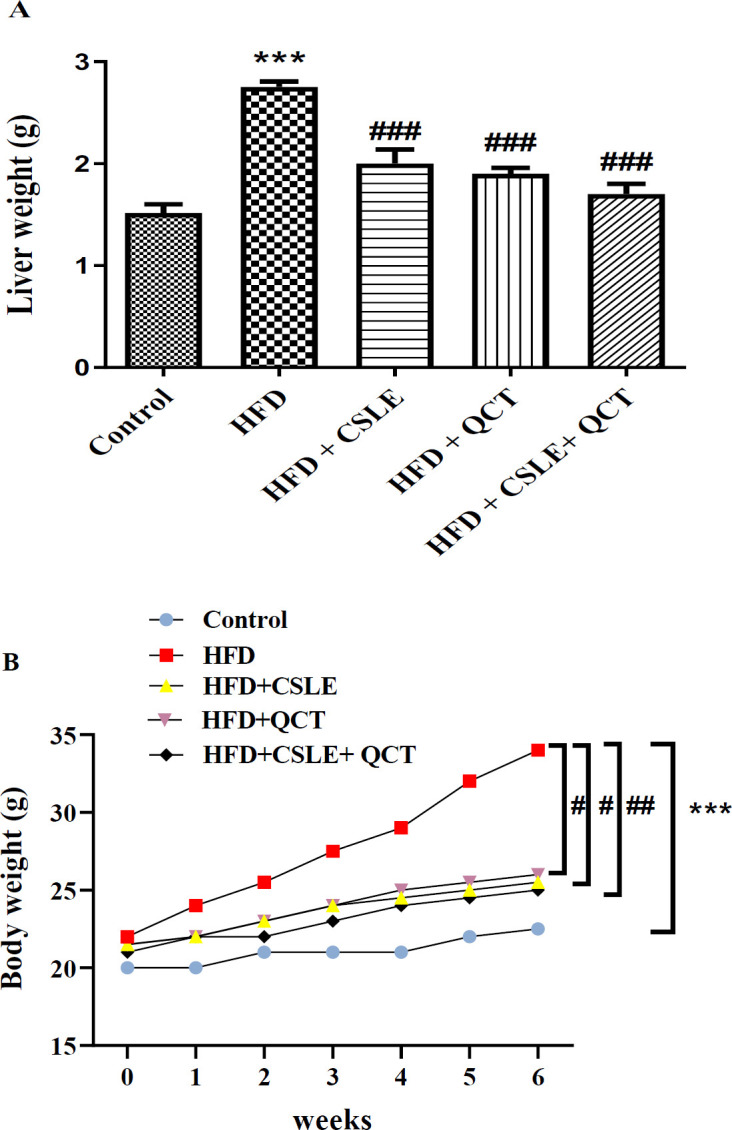
*The effect of* *QCT and* *CSLE on (A) liver weight and (B) body* *weight* *in mice fed HFD. Data are expressed as mean ± SEM* *(n=6).* ^***^p<0.001 comparison with the control group. ^#^*p<0.05,* ^##^*p<0.01, and *^###^*p<0.001 comparison of the HFD groups treated with QCT or CSLE with the HFD group.*

As illustrated in Figure 1, the liver weight and body weight of the HFD group were markedly higher than the control group (P<0.001). In contrast, the HFD group that received treatment with CSLE, QCT, or their combination had lower liver weight and body weight than the HFD group.

### The effect of QCT and CSLE on the liver function markers

**Figure 2:**
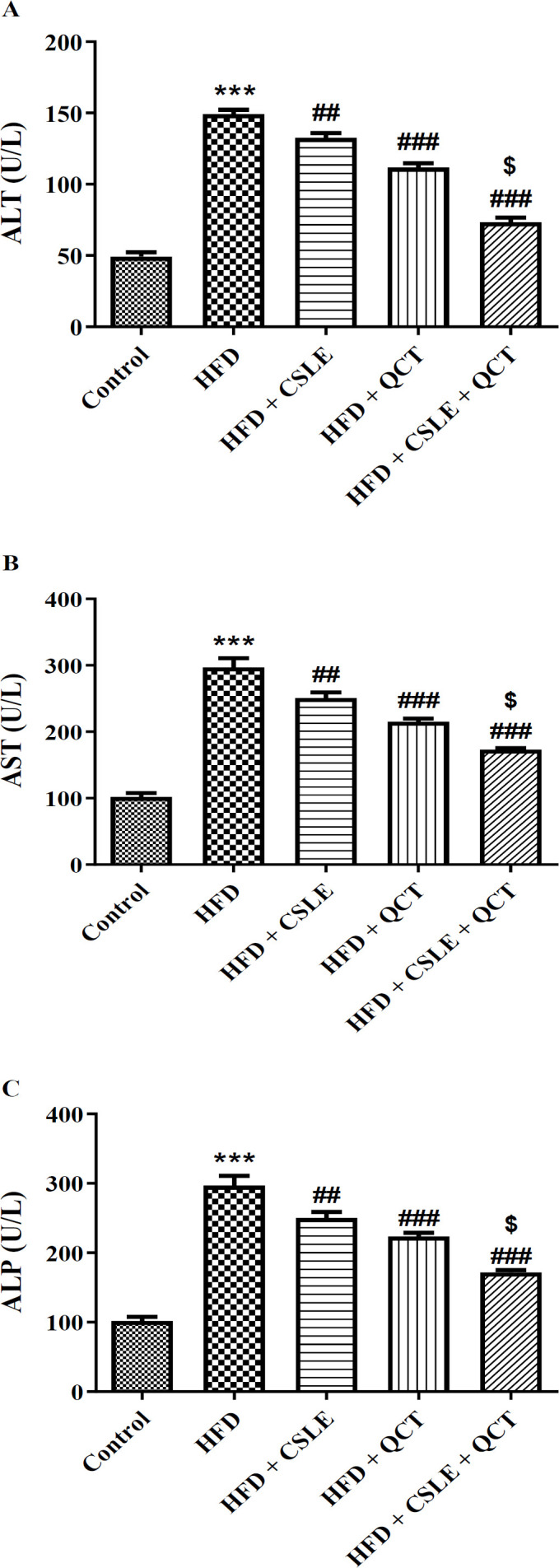
*The effect of* *QCT and* *CSLE on the liver function enzymes (A) ALT, (B) AST, and (C) ALP in mice fed with HFD. Data are expressed as mean±SEM* *(n=6).* ^***^p<0.001 comparison with the control group. ^##^*p<0.01 and *^###^*p<0.001 comparison of the HFD groups treated with QCT or CSLE with the HFD group. *^$^*p<0.05 comparison of the HFD group treated with combination of QCT and CSLE with the HFD groups treated with QCT or CSLE alone*.

Increased serum levels of ALT, AST, and ALP may indicate liver damage. The findings regarding the activity of these enzymes are illustrated in Figure 2. The consumption of HFD resulted in increased activities of ALT, AST, and ALP compared to the control group (P<0.001). In contrast, treatment with QCT or CSLE led to a significant reduction in the activities of these enzymes compared to the HFD group (P<0.001). This study demonstrated that the combination of QCT and CSLE was more effective than either QCT or CSLE alone in ameliorating fatty liver in mice.

### The effect of QCT and CSLE on the lipid profile

**Figure 3:**
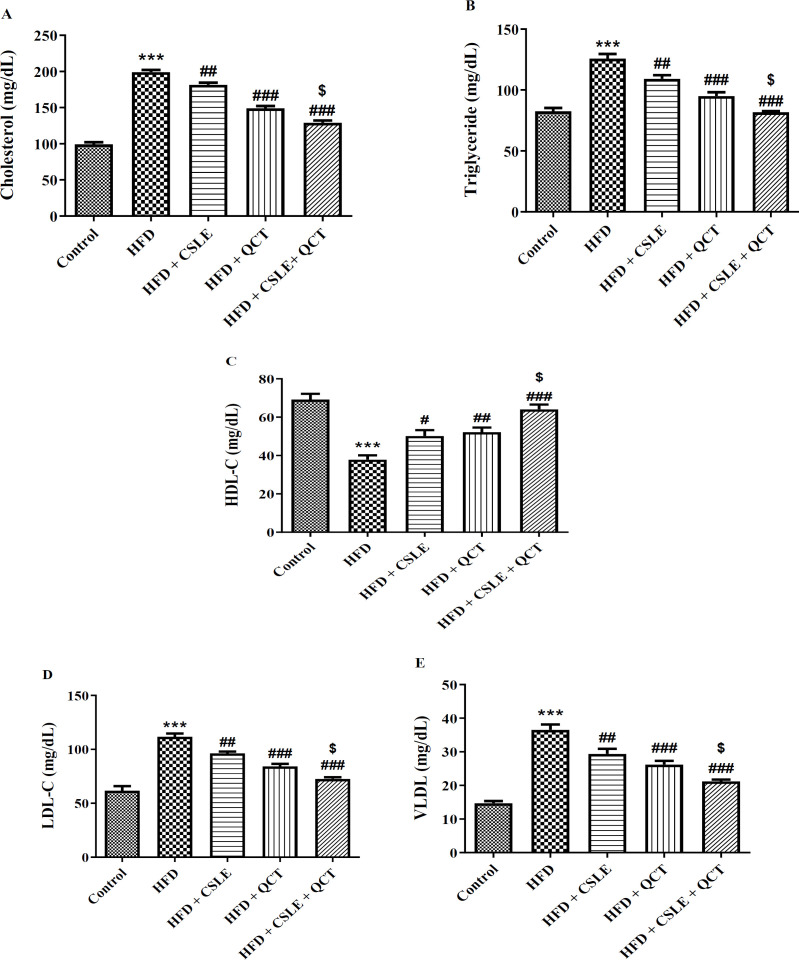
*The effect of* *QCT and* *CSLE on* *the* *levels of lipid profile (A) cholesterol, (B) triglyceride, (C) HDL-C, (D) LDL-C, and (E) VLDL in mice fed HFD.* *Data are expressed as mean ± SEM (n=6).* ^***^p<0.001 comparison with the control group. ^#^*p<0.05, *^##^*p<0.01, and *^###^*p<0.001 comparison of the HFD groups treated with QCT or CSLE with the HFD group. *^$^*p<0.05 comparison of the HFD group treated with combination of QCT and CSLE with the HFD groups treated with QCT or CSLE alone.*

The lipid profile results, including cholesterol, triglyceride, HDL-C, LDL-C, and VLDL, are presented in Figure 3. In the groups receiving HFD supplemented with QCT and CSLE, there was a notable reduction in serum levels of cholesterol, triglyceride, LDL-C, and VLDL, alongside an increase in HDL-C level when compared to the HFD group. Furthermore, the group treated with the combination of QCT and CSLE exhibited an additional decrease in cholesterol, triglyceride, LDL-C, and VLDL, as well as a further elevation in HDL-C, relative to the groups receiving either QCT or CSLE alone. Consequently, the combination of QCT and CSLE demonstrated significant effects in ameliorating fatty liver conditions in mice.

### The effect of QCT and CSLE on the total bilirubin and total protein levels

**Figure 4:**
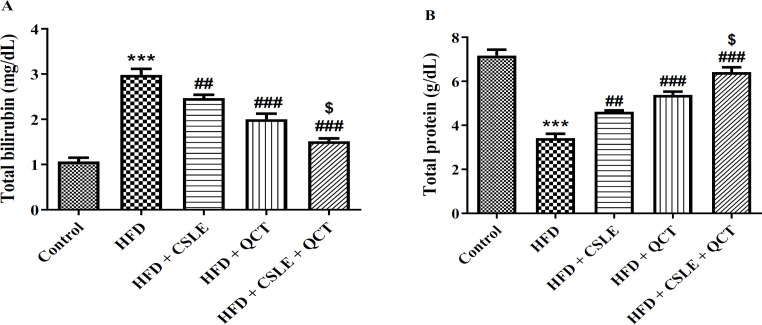
*The effect of* *QCT and* *CSLE on the serum levels of (A)* *T.Bil and* *(B) T.P in mice fed HFD. Data are expressed as mean ± SEM* *(n=6).* ^***^p<0.001 comparison with the control group. ^#^*p<0.05,* ^##^*p<0.01, and *^###^*p<0.001 comparison of the HFD groups treated with QCT or CSLE with the HFD group. *^$^*P<0.05 comparison of the HFD group treated with combination of QCT and CSLE with the HFD groups treated with QCT or CSLE alone.*

Increased serum levels of T.Bil and T.P may indicate liver impairment. The findings regarding T.Bil and T.P levels are illustrated in Figure 4. The administration of HFD resulted in elevated T.Bil and T.P levels compared to the control group (P<0.001). Conversely, treatment with either QCT or CSLE led to a significant reduction in T.Bil and T.P levels compared to the HFD group (P<0.001). The analysis of T.Bil and T.P demonstrated that the combination of QCT and CSLE was more effective in ameliorating fatty liver in mice than either QCT or CSLE administered individually.

### The effect of QCT and CSLE on oxidative stress markers

**Figure 5:**
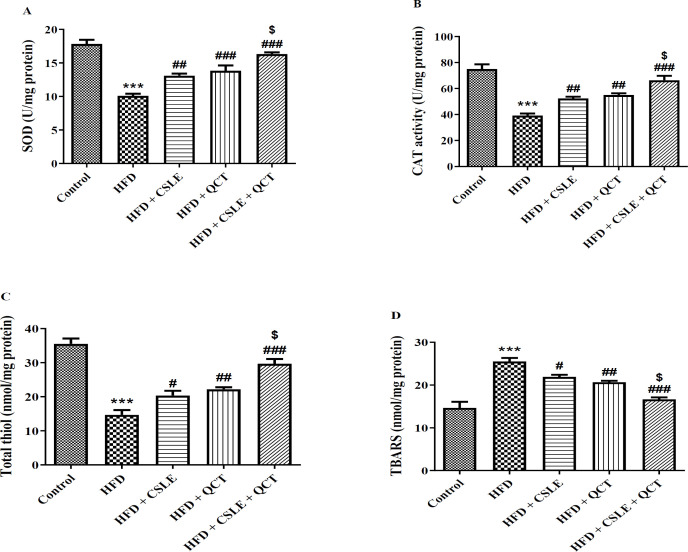
*The effect of* *QCT and* *CSLE on* *oxidative stress parameters (A) SOD, (B) CAT, (C) total thiol, and (D) TBARS in mice fed with HFD. Data are expressed as mean ± SEM (n=6).* ^***^p<0.001 comparison with the control group. ^#^*p< 0.05, (*^##^*p<0.01, and *^###^*p<0.001 comparison of the HFD groups treated with QCT or CSLE with the HFD group. *^$^*p<0.05 comparison of the HFD group treated with combination of QCT and CSLE with the HFD groups treated with QCT or CSLE alone.*

The findings concerning the antioxidant levels of SOD, CAT, total thiol, and oxidant factor TBARS in liver tissue are illustrated in Figure 5. The SOD, CAT, and total thiol levels in the HFD group were markedly lower than those in the control group (P<0.001). Conversely, in the HFD groups that received treatment with QCT or CSLE, the levels of SOD, CAT, and total thiol were significantly elevated compared to the HFD group (P<0.001). The HFD resulted in a notable elevate in TBARS level compared to the control group (P<0.001). However, in the HFD groups treated with QCT or CSLE, there was a significant reduction in TBARS level (P<0.001). The combined effect of QCT and CSLE on oxidative stress markers was significantly more pronounced than the effects observed with either QCT or CSLE administered alone.

### The effect of QCT and CSLE on the level of TNF-α

**Figure 6:**
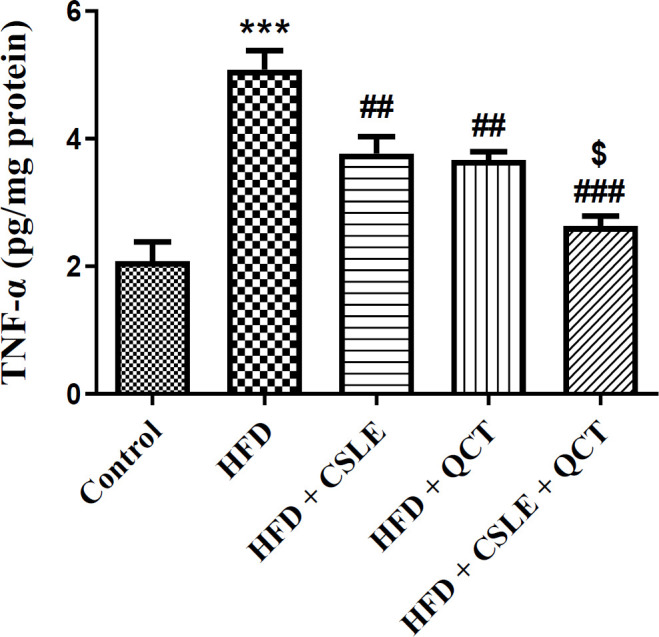
*The effect of* *QCT and* *CSLE on the level of inflammatory cytokine TNF-α in mice fed HFD. Data are expressed as mean ± SEM* *(n=6).* ^***^p<0.001 comparison with the control group. ^#^*p<0.05),* ^##^*p<0.01, and *^###^*p<0.001 comparison of the HFD groups treated with QCT or CSLE with the HFD group. *^$^*p<0.05 comparison of the HFD group treated with combination of QCT and CSLE with the HFD groups treated with QCT or CSLE alone.*

The findings indicated that administration of QCT or CSLE led to a reduction in the hepatic level of the inflammatory factor TNF-α compared to the HFD group. Notably, the HFD group receiving the combined treatment of QCT and CSLE exhibited a more pronounced decrease in TNF-α level than those treated with either QCT or CSLE alone (P<0.001) (Figure 6).

### The effect of QCT and CSLE on histopathological changes

**Figure 7:**
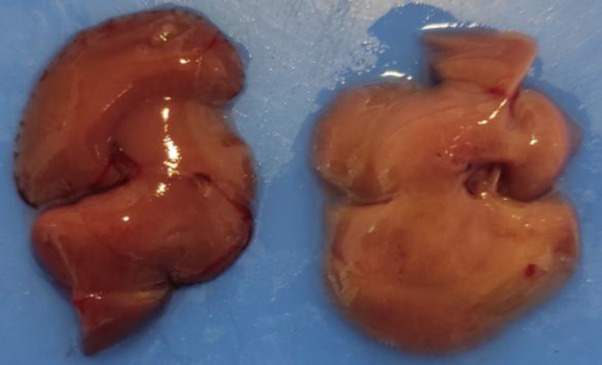
Macroscopic image of normal liver (left) and fatty liver (right).

**Figure 8:**
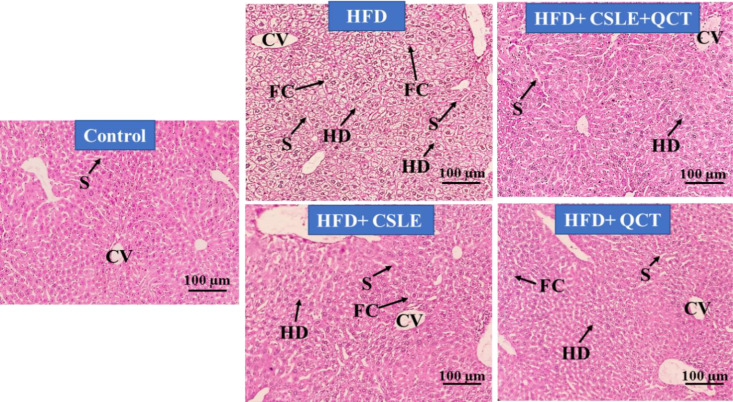
*Hematoxylin-eosin staining of the liver tissue in the treatment groups and the control group. Control group: Liver tissue of mice fed with standard diet,* *HFD group: liver tissue of the mice after six weeks of feeding with* *HFD* *(10 ml/kg),* *HFD+* *QCT group: liver tissue of the mice fed with HFD and then treated with QCT (50 mg/kg), HFD+CSLE group: liver tissue of the mice fed with HFD and then treated with CSLE 2% (200 mg/kg), HFD+* *QCT + CSLE group: liver tissue of the mice fed with HFD and then treated with QCT and CSLE in the last week. Sinusoids (S), lobules disarray, hepatocytes disarray (HD), and fatty change (FC) were observed in the HFD group. In the treated groups, compared to the HFD group, the disarray of the liver lobules was reduced to a large extent, and the FC was significantly less than the HFD group. Magnification: ×100.*

Macroscopic images of the liver from the HFD group (right) and the control group (left) are presented in Figure 7. This image shows a striking contrast between the liver of a HFD-fed mouse and the liver of standard diet-fed mouse. This macroscopic observation aligns with the findings from the microscopic analysis. The microscopic examination of liver tissue stained with H&E for both the treatment groups and the control group is shown in Figure 8. The findings indicated that the liver of the control group exhibited a standard structure characterized by devoid of any indications of damage. Conversely, HFD-fed mice displayed notable histopathological alterations in the liver, including macrovesicular lipid accumulation and swelling of hepatocytes. A semi-quantitative assessment of liver tissue damage is presented in Table 2. In the control group, the liver exhibited no abnormalities. Conversely, in the HFD group, hepatocytes showed obvious changes, with a majority of the cells being filled with adipose tissue. Additionally, these samples demonstrated lobules and hepatocytes disarray (HD), as well as fatty change (FC) within the liver tissue. However, in the HFD groups treated with the combination of QCT and CSLE, the liver tissue showed significantly reduced damage compared to the HFD group, with a notable decrease in fatty change (FC) as well.

**Table 2:** *A semi-quantitative analysis of liver tissue damage in the treatment groups and the control group.* ^**^ *p<0.01 and* ^***^ *p<0.001 comparison with the control group.* ^#^ *p<0.05,* *and* ^###^ *p<0.001* *comparison of the* *HFD* *groups treated with* *QCT* *or CSLE with the* *HFD* *group.*

Groups	Fatty change (%)
**Control**	0.11 ±0.03
**HFD**	4.69 ± 1.13^***^
**HFD + CSEL**	2.21 ± 0.66^**#^
**HFD + QCT**	2.87 ± 0.89^**#^
**HFD + QCT+** ** CSLE**	1.45± 0.35^**##^

**Figure 9:**
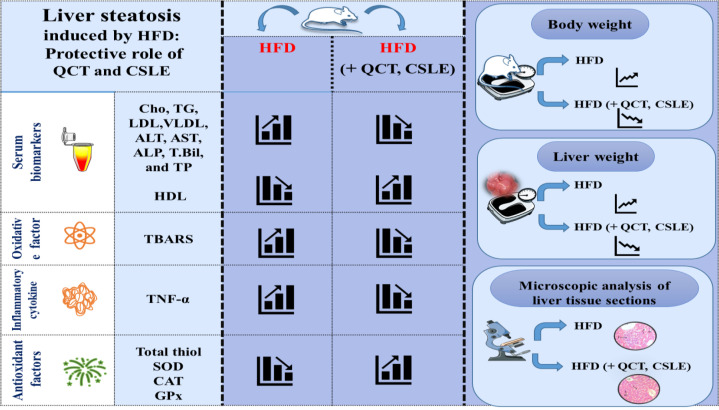
*Graphical abstract shows the protective effects of QCT and* *CSLE* *on the liver steatosis caused by high-fat diet (HFD) in mice. The protective effects of QCT and* *CSLE* *include the reduction of serum biomarkers (Cho, TG, VLDL, AST, ALT, ALP, T. Bil, and T.P) except HDL, the reduction of hepatic TBARS as an oxidative factor, the reduction of TNF‑α as an inflammatory cytokine, increase in antioxidant factors (total thiol, SOD, CAT, and GPx), and improvement of hepatic steatosis caused by HFD.*

## Discussion

NAFLD is a metabolic condition commonly linked to obesity, hyperlipidemia, and type 2 diabetes. Researches indicated that HFD contributes to hepatic steatosis (Assy, et al. 2000). The liver is crucial for fat metabolism, and hepatic steatosis occurs when there is an excessive accumulation of fat within liver cells, resulting from an imbalance between fat synthesis and degradation. 

This research aimed to explore the protective effects of QCT and CSLE against hepatic steatosis induced by HFD, focusing on reducing oxidative stress and inflammation in mice. The findings revealed that HFD led to elevated body weight, liver tissue weight, and serum HDL-C level. Additionally, HFD consumption resulted in elevated ALT, AST, ALP, cholesterol, triglyceride, LDL-C, VLDL, T.P and T.Bil. The HFD also caused an increase in TBARS production while decreasing total thiol level and the activity of antioxidant enzymes such as SOD, GPx, and CAT compared to the control group. The consumption of HFD was associated with heightened energy metabolism, hyperglycemia, and an increase in glucose oxidation, leading to the production of excess oxidative byproducts (van der Kamp and Mulholland 2013). Consequently, the observed reduction in enzymes activity may be attributed to their rapid depletion due to the impaired storage and neutralization of free radicals generated during the HFD feeding period. To assess the therapeutic effects of QCT and CSLE on liver function, the activity of liver enzymes was evaluated. The results indicated a significant reduction in the activity of ALT, AST, and ALP in treatment groups compared to the HFD group, suggesting the protective role of QCT and CSLE on liver function. Previous research has demonstrated that QCT significantly lowers serum transaminase levels, mitigates liver histological alterations, and reduces interleukin-1, interleukin-6, and TNF-α levels, while enhancing cholesterol, triglyceride, SOD, CAT, and total thiol levels in the liver (Albrahim and Alonazi 2021; Ghaeni Pasavei, et al. 2021; Sun, et al. 2024; Yang, et al. 2019), which aligns with our findings. 

Previous research indicates that hypertriglyceridemia and hypercholesterolemia lead to increased serum levels of hepatic enzymes and lipid peroxidation index in liver tissue, while simultaneously reducing antioxidant activity in HFD-fed mice (Amouoghli Tabrizi and Mohajeri 2014; Ding, et al. 2023; Zheng, et al. 2021). In research conducted by Zhou et al., it was found that green tea polyphenols have hepatoprotective properties against various types of liver injury and are proposed as a potential drug candidate for liver diseases (Zhou, et al. 2025). These findings align with our research. Histopathological analysis of liver tissue revealed no indications of damage in the control group. In contrast, HFD-fed mice exhibited notable histopathological alterations, including lobule and HD and FC. Mice treated with QCT and CSLE demonstrated a reduction in both histopathological changes and the extent of liver tissue damage compared to HFD alone-fed mice. This study suggests that QCT and CSLE may be beneficial in ameliorating FLD in HFD-fed mice (Figure 9).

Alkaloids, saponins, flavonoids, phenols, glycosides, tannins, and terpenes represent the active components of CSLE (Al-abodi et al., 2018). A notable limitation is that CSLE should be standardized to gain initial knowledge about possible active ingredients that could elucidate its pharmacological effects.

The current research demonstrated that the combination of QCT and CSLE treatment may significantly diminish liver steatosis in HFD-fed mice by improving lipid profile and antioxidant status. The reduction in serum markers along with the elevation of hepatic antioxidant enzyme activity contributed to the prevention of steatohepatitis development. Ultimately, the administration of QCT and CSLE exhibited a notable impact on the improvement of FLD in HFD-fed mice.
